# A four‐compartment multiscale model of fluid and drug distribution in vascular tumours

**DOI:** 10.1002/cnm.3315

**Published:** 2020-02-25

**Authors:** Rebecca J. Shipley, Paul W. Sweeney, Stephen J. Chapman, Tiina Roose

**Affiliations:** ^1^ Department of Mechanical Engineering University College London London UK; ^2^ Oxford Centre for Industrial and Applied Mathematics Mathematical Institute Oxford UK; ^3^ School of Engineering Sciences, Faculty of Engineering and Physical Sciences University of Southampton Southampton UK

**Keywords:** cancer, chemotherapy, homogenisation, mathematical modelling, vasculature

## Abstract

The subtle relationship between vascular network structure and mass transport is vital to predict and improve the efficacy of anticancer treatments. Here, mathematical homogenisation is used to derive a new multiscale continuum model of blood and chemotherapy transport in the vasculature and interstitium of a vascular tumour. This framework enables information at a range of vascular hierarchies to be fed into an effective description on the length scale of the tumour. The model behaviour is explored through a demonstrative case study of a simplified representation of a dorsal skinfold chamber, to examine the role of vascular network architecture in influencing fluid and drug perfusion on the length scale of the chamber. A single parameter, *P*, is identified that relates tumour‐scale fluid perfusion to the permeability and density of the capillary bed. By fixing the topological and physiological properties of the arteriole and venule networks, an optimal value for *P* is identified, which maximises tumour fluid transport and is thus hypothesised to benefit chemotherapy delivery. We calculate the values for *P* for eight explicit network structures; in each case, vascular intervention by either decreasing the permeability or increasing the density of the capillary network would increase fluid perfusion through the cancerous tissue. Chemotherapeutic strategies are compared and indicate that single injection is consistently more successful compared with constant perfusion, and the model predicts optimal timing of a second dose. These results highlight the potential of computational modelling to elucidate the link between vascular architecture and fluid, drug distribution in tumours.

## Graphical abstract

A novel multiscale continuum model is derived using mathematical homogenisation to predict the delivery of blood and chemotherapy through the vasculature and interstitium of a vascular tumour. Here, the model is used to elucidate the influence of vascular architecture on fluid and drug perfusion in a dorsal skinfold chamber. We identify an optimal permeability and density of the capillary bed, which maximises blood perfusion, and show that single injection of a chemotherapy is more successful than constant perfusion.

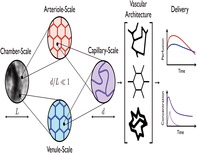

## 1 INTRODUCTION

Solid tumours are characterised by an abnormal microenvironment that distinguishes them from healthy tissue and reduces drug delivery to the cancerous tissue. This is a consequence of numerous factors that include a poorly organised vascular architecture, irregular blood flow, and the compression of blood and lymphatic vessels by cancer cells.^1^ The spatial and temporal heterogeneities in blood supply coupled with variations in the vascular morphology at both microscopic and macroscopic levels cause the spatial distribution of therapeutic agents in tumours to be heterogeneous. Drug treatments (mainly antiangiogenics such as avastin and vascular disrupting and regularising agents such as combretastatins and nelfinavir, respectively) have been developed specifically to target the abnormal vasculature in tumours; however, their impact is hard to predict as the relationship between network structure and the functional parameters that determine mass transport is subtle. For example, increasing the number or diameter of vessels can impair the blood flow distribution, whilst inhibiting angiogenesis is hypothesised to improve circulation^2^; in addition, tumours have been shown to display resistance to vascular disruption therapy via physical mechanisms, such network connectivity and redundancy.^3^

The quality and volume of data characterising tissue vascular architecture is increasing, and it is now possible to describe vascular structure in a highly detailed way. As the resolution of these data continues to increase, it may become too computationally intensive to simulate flow and mass transport in the entire vascular tree using a discrete approach, where vessels are treated individually. In addition, in order to interpret the results, they must not be too sensitive to the full vascular geometry but only to some key characteristics of it. One approach is to employ continuum models in which imaging data are used to deduce functional properties relevant to blood and mass transport.^4^, ^5^, ^6^, ^7^ The mathematical process of homogenisation^8^ is one candidate for developing these continuum models, as it enables spatial heterogeneities at different scales to be transformed into a tractable tissue‐scale model. Macroscale (or tissue‐scale) equations are derived by using a mathematical averaging process to incorporate the relevant microscale topological and model properties. The final macroscale equations can be solved numerically and are far less computationally expensive than solving the microscale equations throughout the domain. A well known example of mathematical homogenisation in practice is the Darcy law; although it was first proposed as an empirical law by Darcy in 1856 (based on water flow experiments through beds of sand),^9^ it was subsequently derived from the Stokes equations using homogenisation techniques.^10^ This homogenisation approach enabled the influence of exact particle shape on hydraulic permeability to be determined.

The hierarchy of healthy vasculature consists of branching arterioles and venules, interconnected by a mesh‐like network of capillaries embedded in the interstitium (composed of cells and extracellular and extravascular space). However, the hierarchical structure of arterioles and venules evident in healthy tissue is disrupted in tumours where it is well‐established that tumour blood vessels are heterogeneous with regard to topology, function, and structure.^11^ Instead, a complex tortuous network of angiogenic blood vessels exists, which are fed and drained by large, often tortuous blood vessels^12^ that results in elevated interstitial fluid pressure^13^ and poor drug penetration to the core of a tumour.^1^, ^3^, ^14^ Here, we use mathematical methods to explore this abnormal tumour microenvironment.

**Figure 1:**
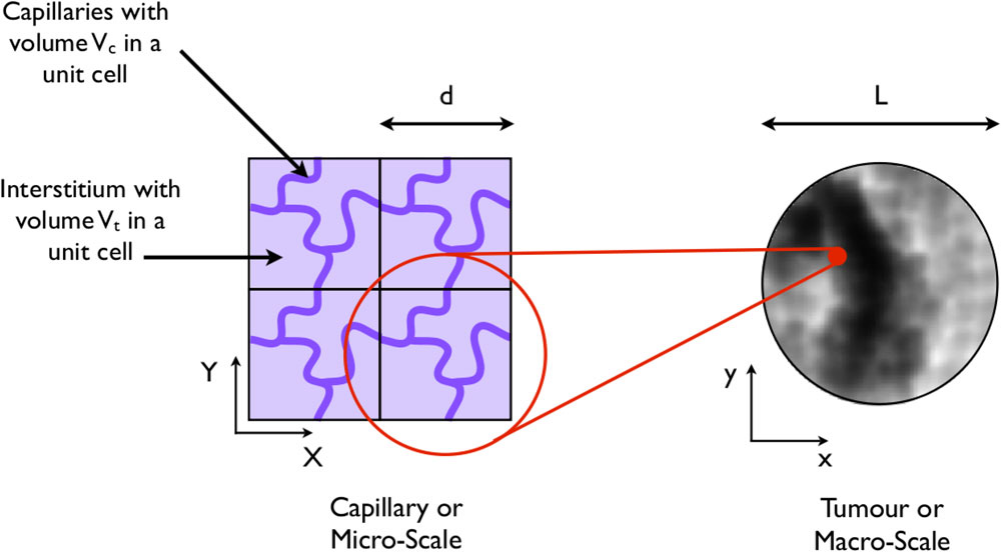
On the left‐hand side is a 2D schematic of a cross‐section through a tissue on the capillary or microlength scale. The capillaries and interstitium are dark and light purple, respectively. A single periodic unit is highlighted in red. The total volume of this unit denotes *V*, whilst the capillary and interstitial volumes are *V*_*c*_ and *V*_*t*_, respectively. The right‐had side depicts the macroscale, where the distribution of fluid and mass appears as a “grey‐scale” and can be modelled as a continuum

The method of homogenisation is applicable to vascular networks with periodic microstructure and with disparate length scales apparent across tissue. This enables us to make progress towards a computationally tractable model whilst retaining key architectural information. The Darcy law has also been shown to provide good predictivity of macroscale fluid mechanics for nonperiodic structures, even if the multiscale derivation assumes periodicity.^15^, ^16^ Figure 1 shows representations of both the capillary or microscale and the tissue‐scale or macroscale. On the microscale, both the capillaries and interstitium are identifiable and are illustrated by periodic changes of dark and light purple regions, respectively. An example periodic unit cell is circled in red, which repeats in all directions (although the structure must be periodic in this sense, there is no other assumption of homogeneity within this unit). The capillary length scale is given by the typical intercapillary distance, *d*. If the ratio between the capillary length scale, *d*, and the tumour or macroscale, *L* is small, that is, *ɛ* = *d*/*L* ≪ 1, it is possible to derive an effective macroscopic model describing both the capillaries and the interstitium. In the schematic on the left‐hand side of Figure 1, the heterogeneities can be distinguished. By comparison, viewed from a distance, or from the tumour or macroscale, the heterogeneities average out as shown in the right‐hand side of Figure 1. Here, both the capillary network and interstitium can be represented as continua; therefore, the distribution of fluid in the two can be represented as a “grey‐scale.” This averaging to a continuum is exactly what the mathematical process of homogenisation achieves. The space variable **x** = (*x*, *y*, *z*) reveals the properties of the system on the length scale of the tumour. Scaling **x** **x** with *ɛ* ^−1^ defines a new space variable **X** = (*X*, *Y*, *Z*) = ɛ^−1^**x**, which reflects the system properties at the capillary scale. One of the fundamental assumptions of homogenisation is that *ɛ* is sufficiently small that the length variables **x** and **X** are disparate and can be treated as two independent variables.^8^

In this paper, we use multiscale mathematical models to investigate the dependence of fluid perfusion and drug distribution properties on vascular structure under two key assumptions. First of all, we discretise the vascular tree according to vessel dimension, although in reality, the topological properties of healthy vessels are continuously distributed from the arteries to the capillary bed and to the veins. We divide the vascular tree into a supplying artery and draining vein, together with arteriole, venule, and capillary networks, and categorise these networks by three different length scales. These are the length scale of the tissue, the arterioles and venules (a typical interarteriole or intervenule separation) and the capillaries (a typical intercapillary separation). In this way, the dependence of tumour‐scale fluid and drug perfusion properties on vasculature structure can be seen as occurring through the interaction of the various length scales under consideration.

Second, we assume that the structure is spatially periodic on the capillary, arteriole, and venule length scales. For example, on the length scale of the capillaries, the tumour is composed of a periodic array of capillaries embedded in the interstitium, as depicted in Figure 1. Although this is a simplifying assumption, the hierarchical vascular structure evident in healthy tissue rarely occurs in vascular tumours.^11^, ^12^ Therefore, we assume an unstratified, periodic tumor vasculature enabling continuum mathematical models to be derived using homogenisation. These continuum models are computationally tractable compared with simulations throughout the vascular tree and enable the impact of varying vascular structure on tumour‐scale fluid perfusion and mass transport to be tested, without re‐deriving the models each time. In the long term, this will help to both elucidate the mechanisms underlying transport and to quantify the impact of vascular structure on tumour‐scale fluid and drug perfusion.

In previous studies,^17^, ^18^ we used homogenisation to derive effective fluid and drug perfusion models for a capillary bed and surrounding interstitium. The final fluid equations comprised a double porous medium, with coupled Darcy flow through the interstitium and vasculature, whereas the drug equations comprised advection‐reaction equations; in each case, the dependence of the transport coefficients on the vascular geometry was determined by solving capillary‐scale cell problems. In this paper, we extend that approach to a four‐component tissue structure, so that vascular hierarchy may be accounted for in the models. We derive a new set of effective equations for fluid and drug distribution and explore the role of vascular architecture in determining tissue‐scale fluid and drug perfusion using an example case study of a rodent model for monitoring of microvascular pathophysiology in vivo (a dorsal skinfold chamber).

## 2 THE MODEL SET‐UP

**Figure 2:**
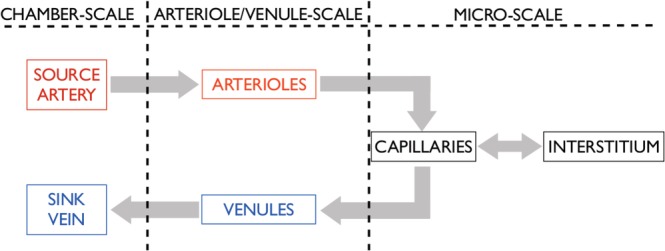
The fluid pathway. Fluid flows from a source artery, through the arterioles into the capillaries, and then out of the chamber through a sink vein via the venules. Since the capillaries are leaky, there may also be fluid exchange between the capillaries and the interstitium

A dorsal skinfold chamber is the chronic implantation of an observation chamber on the dorsal skinfold of rodents, which enables repeated, intravital microscopic observations.^19^ These chambers are a common tool for therapy development, particularly for cancers. In this paper, we use a simplified representation of this chamber structure as a case study to explore implementation of the four‐component transport model and to explore the role of vascular architecture in determining effective parameters relating to fluid and drug delivery. Blood flow through a chamber containing cancerous tissue is captured by considering four separate components: the arteriole, venule and capillary networks, and the interstitium (composed of cells and extracellular and extravascular space). We assume that fluid flows from a seeded feeding artery, through the arterioles into the capillaries, then into the venules before leaving the chamber via a seeded draining vein. Since the capillaries are leaky, there may also be fluid exchange between the capillaries and the interstitium. In our model, it is not possible for fluid to travel directly from the arterioles into the venules bypassing the capillaries. The fluid pathway is summarised in Figure 2. Throughout this paper, we assume that dorsal skinfold chambers do not have functioning lymphatics because of clamping of the chamber to the skin and general lack of lymphatics in tumours and therefore neglect this aspect in the fluid transport models. Further, whilst difficulties exist in categorising blood vessels into distinct groups,^12^ for convenience, we divide the tumour vasculature into three separate components: arteriole, capillary, and venule networks.

The model structure explained above can be described using three different length scales. The finest scale is referred to as the “microscale” or the “capillary length scale” and describes the capillaries and interstitium, characterised by a typical intercapillary separation *d* ≈ 50 μm. The most course scale is referred to as the “chamber scale” and describes the scale of the entire chamber, that is, *L* ≈ 10^4^ μm = 1 cm. In between these scales, we introduce a third length‐scale, which relates to the separate arteriole or venule networks, characterised by a typical vessel separation *s* ≈ 10^3^ μm. These three length scales are depicted in Figure 4 and interact through two dimensionless ratios

(1) $$\nu =\frac{d}{s}\approx 5\times 10^{-2},\eta =\frac{s}{L}\approx 10^{-1}.$$

On the chamber scale, none of the arterioles, venules, capillaries, or interstitium are distinguishable; however, a seeded artery and vein supply a pressure gradient across the vasculature. Zooming into any point on the chamber scale reveals the arteriole and venule scales. Here, blood flows through the arterioles or venules and seeps into a porous tissue matrix composed of capillaries and interstitium. Finally, zooming into a point in the tissue on the length scale of the arterioles and venules reveals the capillary scale. Here, blood flows through the capillary network and seeps into the interstitium.

As *ν* ≪ 1, the micro and arteriole/venule length scales are well‐separated. Therefore, it is possible to use asymptotic homogenisation to move from the micro to the arteriole/venule descriptions and derive the porous medium equations for the capillary network and interstitium on the length scale of the arterioles and venules. Similarly, *η* ≪ 1, so we can move from the arteriole/venule to the chamber descriptions and derive the coupled porous medium equations that describe fluid transport in the arterioles, venules, capillaries, and interstitium on the scale of the chamber.

Next, we consider fluid and drug transport in turn. We present and analyse the fluid and drug transport problems on the length scale of the chamber, as well as a summary of the derivation steps using homogenization, including parameter estimation on each of the length scales described above.

### 2.1 Fluid transport model

We denote the fluid pressure by *p* and the velocity by **u**, with subscripts *a*, *v*, *c*, or *t* denoting the values of *p* or **u** in the arterioles, venules, capillaries, and interstitium, respectively.

#### 2.1.1 Fluid transport on the scale of the capillaries

The capillaries of a tumour are embedded in the interstitium, which is itself composed of cells and extracellular space. We assume the tumour occupies a three‐dimensional volume and can be described as spatially periodic on the capillary scale. This structure is depicted by a 2D schematic in Figure 1, where a single periodic unit cell is highlighted in red. We denote the total volume of a unit cell by *V*, the volume of the capillaries in a unit by *V*_*c*_, and the volume of the interstitium by *V*_*t*_. We denote the fluid velocity by **u** and the pressure by *p*, with subscripts *t* or *c* denoting the interstitium or capillaries, respectively. We assume that the flow in both the interstitium and the capillaries is incompressible. The interstitium is composed of cells surrounded by extracellular space. However, the capillaries are much larger than the intercell separation of the interstitium, and so we treat the interstitium as an isotropic porous medium and describe fluid flow through it by the Darcy law (eg, previous studies^20^, ^21^). Therefore, in the interstitium,

(2) $$\nabla \cdot u_{t}=0,u_{t}=-\frac{k^{\mathrm{int}}}{\mu }\nabla p_{t},$$

where *k* ^*int*^ is the interstitial permeability and *μ* is the viscosity of blood plasma.

Blood flow in the capillaries of the microcirculation is a highly complex process. Healthy human blood is a concentrated suspension containing red blood cells (RBCs) at a concentration (haematocrit) of 40% to 45%. In vessels much larger than the RBCs (ie, with diameter much larger than ≈ 8 μm), blood can be treated as a continuum with a viscosity that is approximately constant. In vessels smaller than this, the finite size of RBCs results in noncontinuum behaviour and complex rheology that causes several important effects, for example, the Fåhraeus^22^ and Fåhraeus‐Lindqvist^23^, ^24^ effects, and phase separation at diverging bifurcations.^25^, ^26^ Empirical laws have been developed to describe these non‐Newtonian effects,^24^, ^26^ and these have also been incorporated into homogenisation frameworks (eg, Penta et al^27^). The introduction of more realistic haemodynamic descriptions of blood are important to predict tissue‐scale fluid mechanics and would result in an effective and spatially dependent tissue‐scale viscosity.

Because of the additional computational expense of incorporating non‐Newtonian haemodynamics, and the emphasis here on the development and application of a new modelling framework, we neglect non‐Newtonian effects here and assume that the fluid flow in the capillaries is described by the Navier‐Stokes equations for a fluid of constant viscosity. Therefore, in the capillaries, we have

(3) $$\nabla \cdot u_{c}=0,\rho \left(\frac{\partial u_{c}}{\partial t}+\left(u_{c}\cdot \nabla \right)u_{c}\right)=-\nabla p_{c}+\mu \nabla^{2}u_{c},$$

where *ρ* is the fluid density. The leakage from the capillaries into the interstitium is given by the Starling law, **q**_*e*_ = *L*_*p*_(*p*_*c*_ − *p*_*t*_)**n**, where **q**_*e*_ is the leakage flux, *L*_*p*_ is the vascular permeability (assumed constant), **n** is the unit outward pointing normal to the capillary surface, and *p*_*c*_, *p*_*t*_ are evaluated on the interior and exterior sides of the capillary wall, respectively. Given this, we impose continuity of mass flux across the capillary walls, so that

(4) $$u_{t}\cdot n=u_{c}\cdot n=L_{p}\left(p_{c}-p_{t}\right)\;\mathrm{on}\;\text{the capillary walls}.$$

Finally, we impose a no‐slip condition on the capillary walls so that

(5) $$u_{c}\cdot \tau =0\;\mathrm{on}\;\text{the capillary walls},$$

where ***τ*** is a unit tangential vector to the capillary wall. In practice, there is slip at the capillary surface, and it is determined by the microvascular rheology, in particular, the structure of the endothelial glycocalyx. Given that we have neglected non‐Newtonian effects here, it is sensible to simplify to the no‐slip boundary condition (5).

#### 2.1.2 Parameter estimation

##### Geometrical parameters

Geometrical data on capillaries are presented in Konerding et al,^28^, ^29^ with data on the capillaries, arterioles, and venules in a dorsal skinfold chamber summarised in Table 1. A typical intercapillary distance *d* is 50 μm, whilst a representative mean interarteriole or intervenule distance *s* is 10^3^μm. This gives a typical value of *ν* as 5 × 10^−2^, justifying the assumption *ν* ≪ 1.

**Table 1:** A table of the fixed parameters

Parameter	Value	Units	Formula	Description
**Geometrical and physiological parameters**				
*s*	100	μm	/	Arteriole and venule length scale
*L*	1	cm	/	Chamber length scale
*d*_2_	50	μm	/	Capillary length scale (region 2)
*μ*	4 × 10^−3^	kg/m/s	/	Viscosity of blood
*k* ^*int*^	0.4	μm^2^	/	Permeability of the interstitium
*R*_*a*_ = *R*_*v*_	10^−2^	/	/	“Leakiness” of the arterioles and venules
*k*_2_	37.4	μm^2^	/	Permeability of the region 2 capillary network
*h*	37.4	μm^2^	/	Permeability of the arteriole and venule networks
*S*_*a*_/*V*_*a*_ = *S*_*v*_/*V*_*v*_	6.47 × 10^−4^	μm^−1^	/	Surface area to volume ratio
*S*_*a*_/*V*_*p*_ = *S*_*v*_/*V*_*p*_	9.86 × 10^−5^	μm^−1^	/	Surface area to volume ratio
**Length scale ratios**				
*ν*	5 × 10^−2^	/	*d*/*s*	Length scale ratio
*η*	0.1	/	*s*/*L*	Length scale ratio
**Modelling parameters**				
*n*	0.152	/	*V*_*a*_/*V*_*p*_	Volume ratio
*r*	0.986	/	*μL*_*a*_ *L* ^4^ *S*_*a*_/*V*_*p*_ *s* ^4^	Modelling ratio
*C*	4 × 10^−9^	m^2^	*k* ^*int*^ *L* ^2^/*s* ^2^	Modelling ratio
*P*_mal_	9.36 × 10^−12^	m^2^	*k*_mal_ *L* ^2^ *d*_mal_^2^/*s* ^4^	Modelling ratio

##### Physiological parameters

Experimental values of the hydraulic conductivity *k* ^*int*^/*μ* are determined in previous studies^30^, ^31^, ^32^ for rat squamous cell tissue, hepatoma *in vitro*, mouse mammary carcinoma, and rat hepatocarcinoma tissue; values lie in the range 10^−9^ to 10^−6^ cm^3^ s kg^−1^. However, these values are obtained by applying the Darcy law to *in vitro* filtration data; measuring the hydraulic conductivity *in vivo* is very difficult. The blood viscosity, *μ*, depends on the hematocrit (the density of RBCs) and the temperature and (though we are approximating blood as a Newtonian fluid) also on the shear rate. Nevertheless, for a normal 40*%* hematocrit and 37°C, *μ* ≈ 4 × 10^−3^ kgm^−1^ s^−1^.^33^ On this basis, the interstitial permeability *k* ^*int*^ lies between 4 × 10^−14^ and 4 × 10^−11^ cm^2^; although the range in practice is likely to be much larger and highly dependent on the tissue type.

The vascular hydraulic permeability *L*_*p*_ can be difficult to estimate. In Sevick and Jain,^34^ the authors attempt to address this by measuring the capillary filtration coefficient. Using this technique, values for *L*_*p*_ can be extracted from the data in previous studies^31^, ^35^ for mouse mammary carcinoma and healthy rat hindquarter tissue and are about 10^−6^ cm^2^ s kg^−1^. Finally, the blood density *ρ* ≈ 1040 kg m^−3^,^36^ and we assume a typical velocity in the capillaries is *U* ≈ 25 μm s^−1^.

There are three dimensionless parameters that characterize the fluid transport problem. First of all, the Reynolds number, Re = *ρUd*/*μ* represents the ratio of inertial to viscous forces in the capillaries. Therefore, if inertia dominates over viscosity, Re is large, whereas if viscosity dominates over inertia, Re is small. Here, we find that Re ≈ 3.3 × 10^−4^, and therefore, the importance of inertia is negligible on the microscale. The final two dimensionless parameters are

(6) $$\kappa =\frac{k^{\mathrm{int}}s}{d^{3}}\;\text{and}\;R=\frac{\mu L_{p}s^{3}}{d^{4}},$$

which represent the relative permeability of the interstitium and capillary walls, respectively, in comparison with fluid transport. We find that *R* ≈ 6.4 × 10^−5^ and *κ* lies in the range 3.2 × 10^−8^ to 3.2 × 10^−5^. The relative sizes of these parameters are important for the homogenisation analysis. Specifically, the average fluid velocity on the length scale of the arterioles and venules will be dominated by that in the capillaries, and the contribution of the interstitium to the average fluid velocity is of size *ν*.

#### 2.1.3 Fluid transport on the scale of the arterioles and venules

We homogenise the model for fluid transport in the capillaries and interstitium, given by Equations (2) to (5), to give a description for transport in the capillaries and interstitium on the scale of the arterioles and venules. The homogenisation steps are detailed in previous studies^18^, ^37^ and may be summarised as (a) assuming length scale separation between the micro and arteriole/venule length scales by substituting ∇ = ∇_micro_ + *ν*∇_arteriole/venule_ in Equations (2) to (5), (b) expanding all variables asymptotically in powers of *ν*, (c) equating coefficients of powers of *ν* in (2) to (5), (d) decomposing the fluid velocity **u** into components that vary on the microscale and arteriole/venule scales, with the component relating to the micro problem being defined through a cell problem on the microscale geometry, (e) averaging over the microscale to derive the arteriole/venule scale relationships. On the arteriole/venule length scale, the capillaries and interstitium appear as continua and behave as a double porous medium with coupled Darcy flow between the two. The equations for fluid transport in the capillaries and interstitium, on this length scale, are

(7) $$u_{c}=-\frac{\eta }{\mu }K\cdot \nabla p_{c},\;\nabla \cdot \left(K\cdot \nabla p_{c}\right)=0,u_{t}=-\frac{\nu \eta }{\mu }E\cdot \nabla p_{t},\;\nabla \cdot \left(E\cdot \nabla p_{t}\right)=\frac{\mathrm{RSd}}{V_{t}}\left(p_{t}-p_{c}\right),$$

where *R* (which is dimensionless) represents the leakiness of the capillary walls (and is given in 6) and *S*/*V*_*t*_ is the ratio of the surface area of the capillaries to the volume of interstitium. Further, **K** and **E** are the fluid permeability tensors associated with the capillary network and interstitium and are determined by the homogenisation process. They are given explicitly at the end of this section in terms of two cell problems that must be solved on the length scale of the capillaries, once the capillary‐scale geometry has been specified. It is precisely these permeability tensors **K** and **E** that relate transport on courser length scales to the geometry and transport properties on the capillary scale.

**Figure 3:**
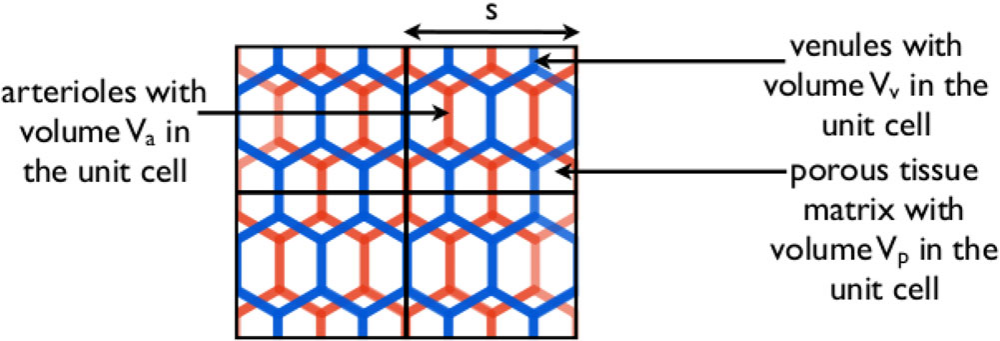
A schematic of a cross‐section through the tumour on the length scale of the arterioles and venules. The arterioles, venules, and porous tissue matrix (composed of the capillary bed and interstitium) are in red, blue, and white, respectively. The arteriole, venule, and porous tissue volumes in a unit cell are denoted *V*_*a*_, *V*_*v*_, and *V*_*p*_, respectively

On the length scale of the arterioles and venules, fluid flows from the arterioles into the porous tissue matrix (composed of the capillary network and interstitium) and then into the venules; a schematic of this set‐up is shown in Figure 3. We denote the volume of arterioles and venules in a unit by *V*_*a*_ and *V*_*v*_, respectively, and the volume of porous tissue matrix by *V*_*p*_. Equation (7) describes the fluid transport in the porous tissue matrix. We use subscripts *a* and *v* to denote the arterioles and venules, respectively, and describe blood flow in the arterioles and venules using the Navier‐Stokes equations so that

(8) $$\nabla \cdot u_{i}=0,\rho \left(\frac{\partial u_{i}}{\partial t}+\left(u_{c}\cdot \nabla \right)u_{i}\right)=-\nabla p_{i}+\mu \nabla^{2}u_{i},$$

where *i* = *a*, *v* distinguishes between the arterioles and venules. Fluid flows from the arterioles into the capillary bed (and from the capillary bed into the venules) because of a pressure drop across the vascular tree. Therefore, on the boundaries of the arterioles and porous tissue matrix, and the venules and porous tissue matrix, we impose the boundary conditions

(9) $$u_{a}\cdot n_{a}=u_{c}\cdot n_{a}=L_{a}\left(p_{a}-p_{c}\right),\;u_{v}\cdot n_{v}=u_{c}\cdot n_{v}=L_{v}\left(p_{v}-p_{c}\right),$$

respectively, where *L*_*a*_ and *L*_*v*_ represent the “leakage” of fluid from the arterioles into the porous tissue matrix, and from the porous tissue matrix into the venules, respectively, and **n**_*a*_, **n**_*v*_ are the unit outward pointing normals to the arteriole and venule boundaries, respectively. Finally, to close the model, we impose no slip on the arteriole/porous tissue matrix and venule/porous tissue matrix boundaries, so that **u**_*a*_·***τ***_*a*_ = 0 and **u**_*v*_·***τ***_*v*_ = 0 on the appropriate boundaries, where ***τ***_*a*_, ***τ***_*v*_ are the unit tangential vectors to the arteriole and venule boundaries.

**Figure 4:**
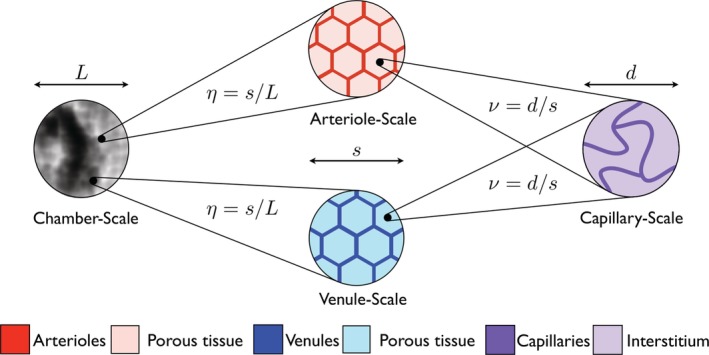
The three characterizing length scales are the microscale of the capillaries (*d* ≈ 50 *μ*m), the arteriole/venule scale (*s* ≈ 10^3^ *μ*m), and the chamber scale (*L* ≈ 10^4^ *μ*m). These three length scales interact through the dimensionless ratios *ν* = *d*/*s* and *η* = *s*/*L*

**Figure 5:**
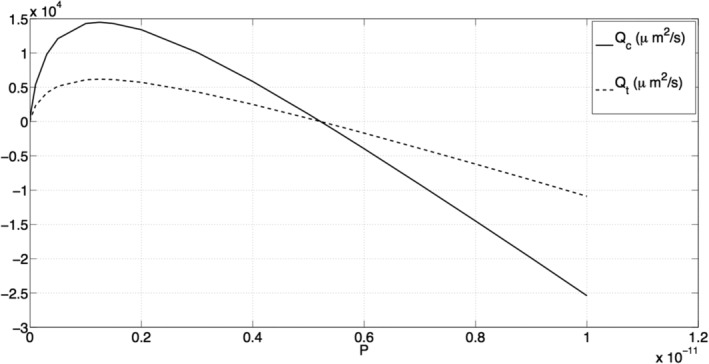
A plot of *Q*_*c*_ and *Q*_*t*_ (both measured in μm^2^ s^−1^) against the parameter *P* (measured in m^2^) that captures the permeability and density of the capillary network

**Figure 6:**
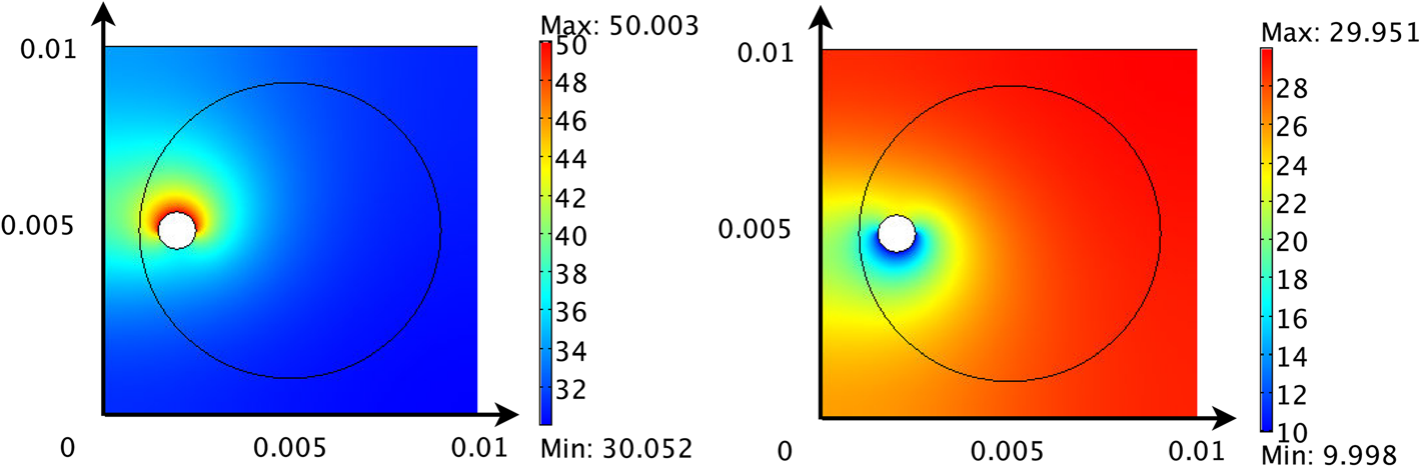
The solutions for *p*_*a*_ and *p*_*v*_ when *P* = 1 × 10^−14^ m^2^. Chamber dimensions are metres, and all pressures are given in units of mmHg

**Figure 7:**
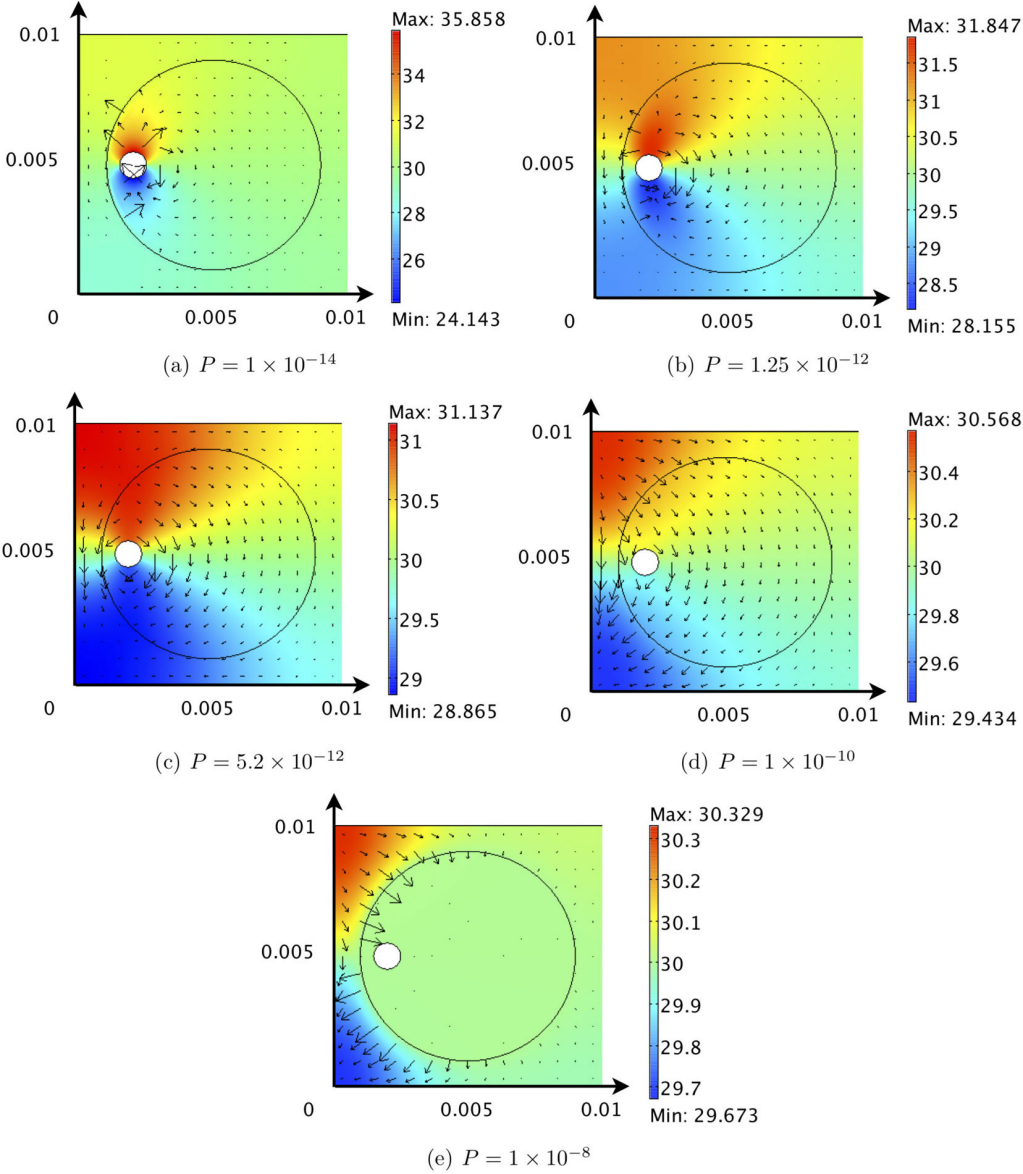
The solutions for *p*_*c*_ = *p*_*t*_ for various values of *P*. Chamber dimensions are metres, and the arrows represent **u**u_*c*_ (arrow length is proportional to the magnitude of the velocity vector). All pressures are given in units of mmHg

**Figure 8:**
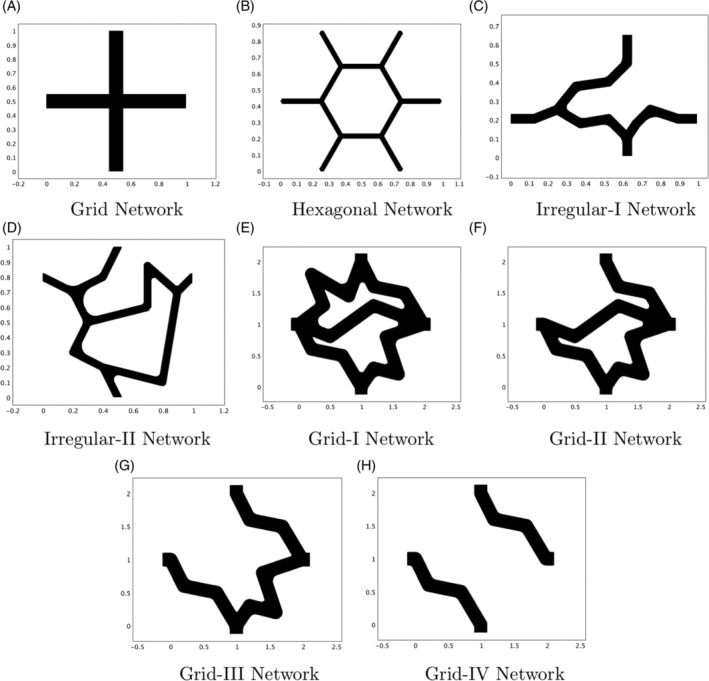
Some explicit network examples

**Figure 9:**
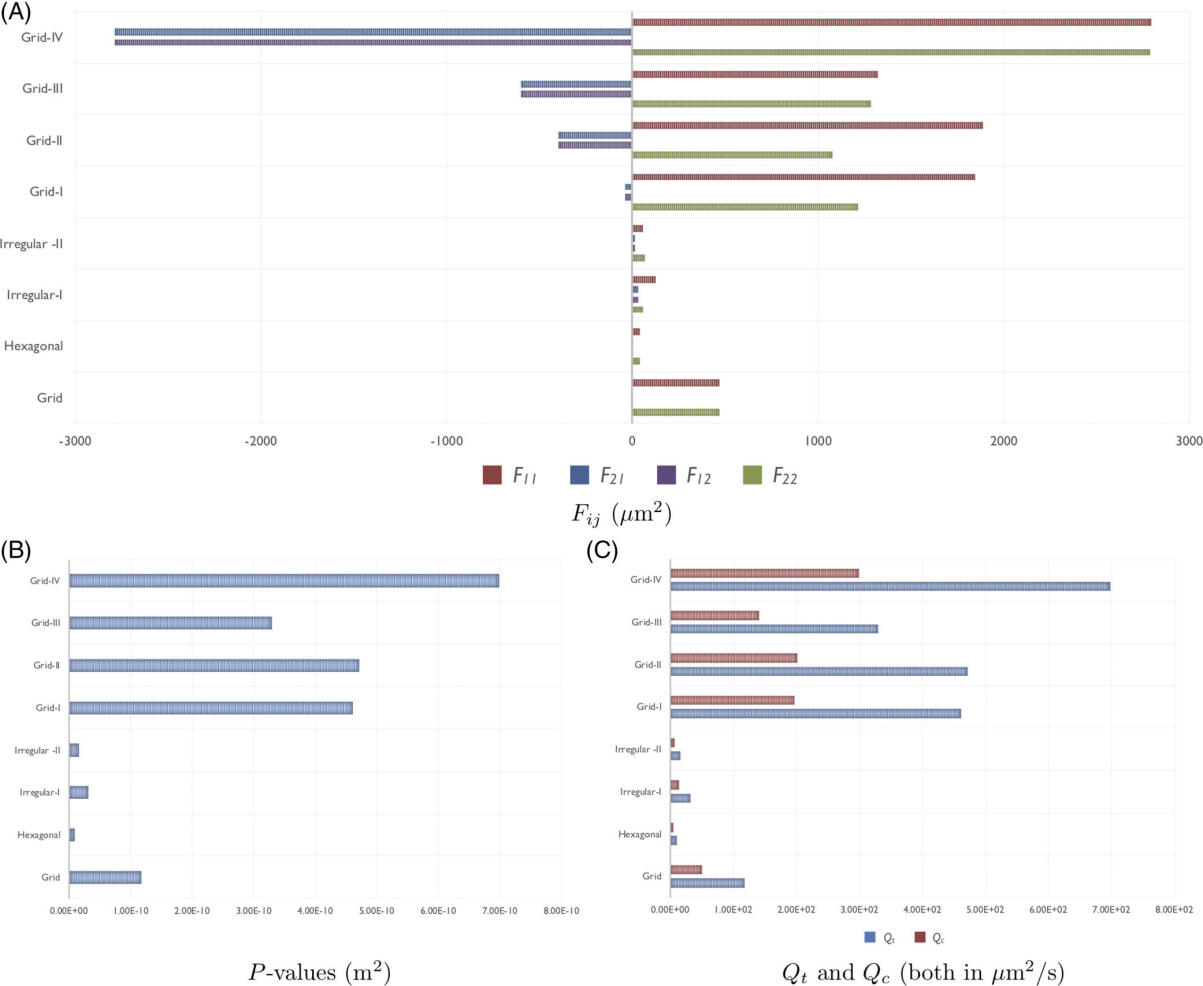
The *P* values and corresponding values of *Q*_*c*_ and *Q*_*t*_ for each of the explicit networks of Figure 8

**Figure 10:**
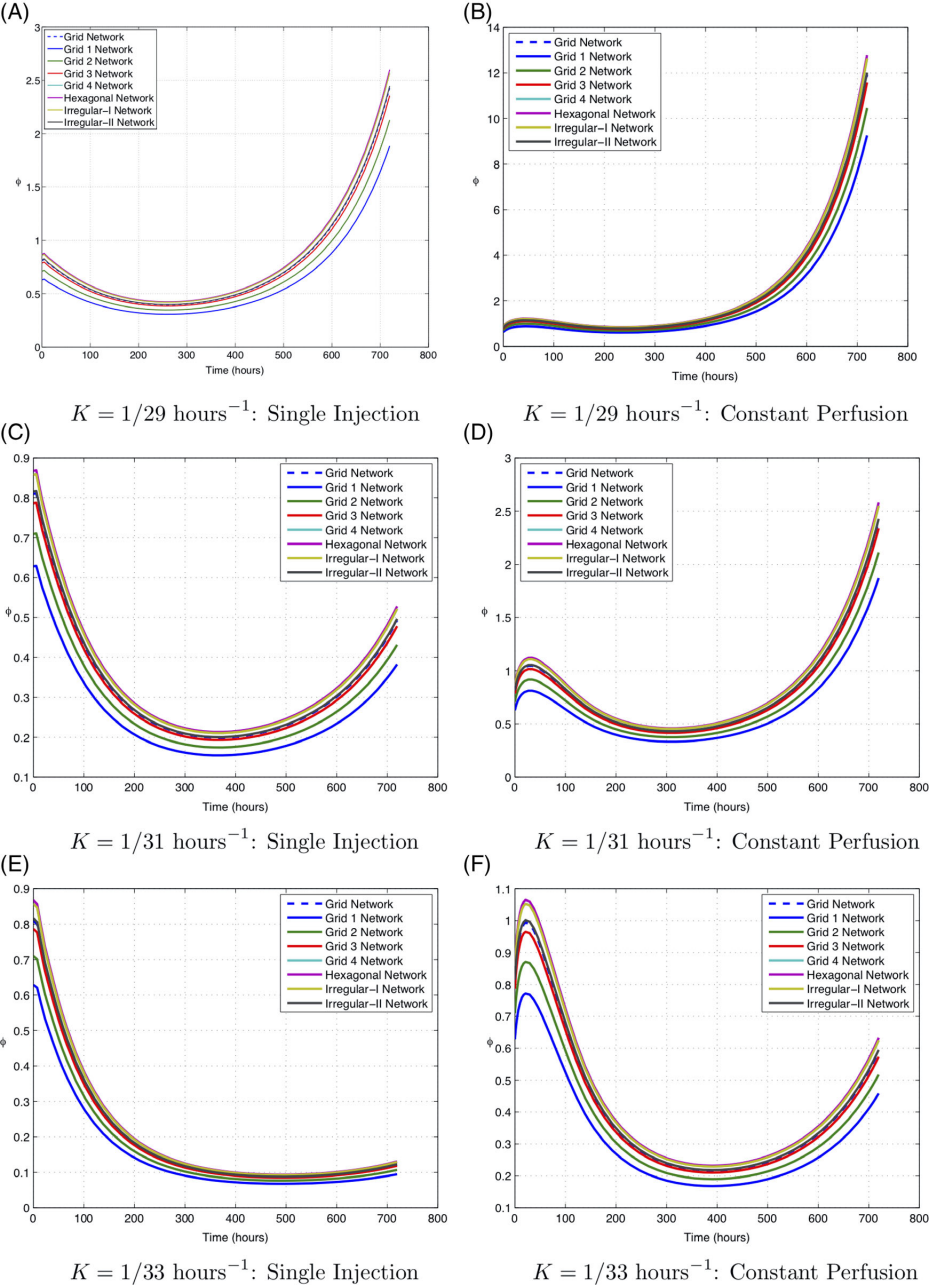
The variation of the average interstitial volume fraction as a function of time

**Figure 11:**
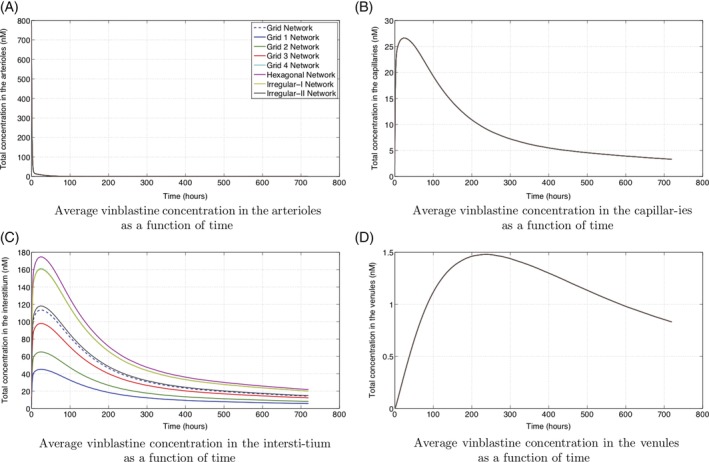
The average vinblastine concentration in each of the arterioles, capillaries, interstitium, and venules for a single injection

**Figure 12:**
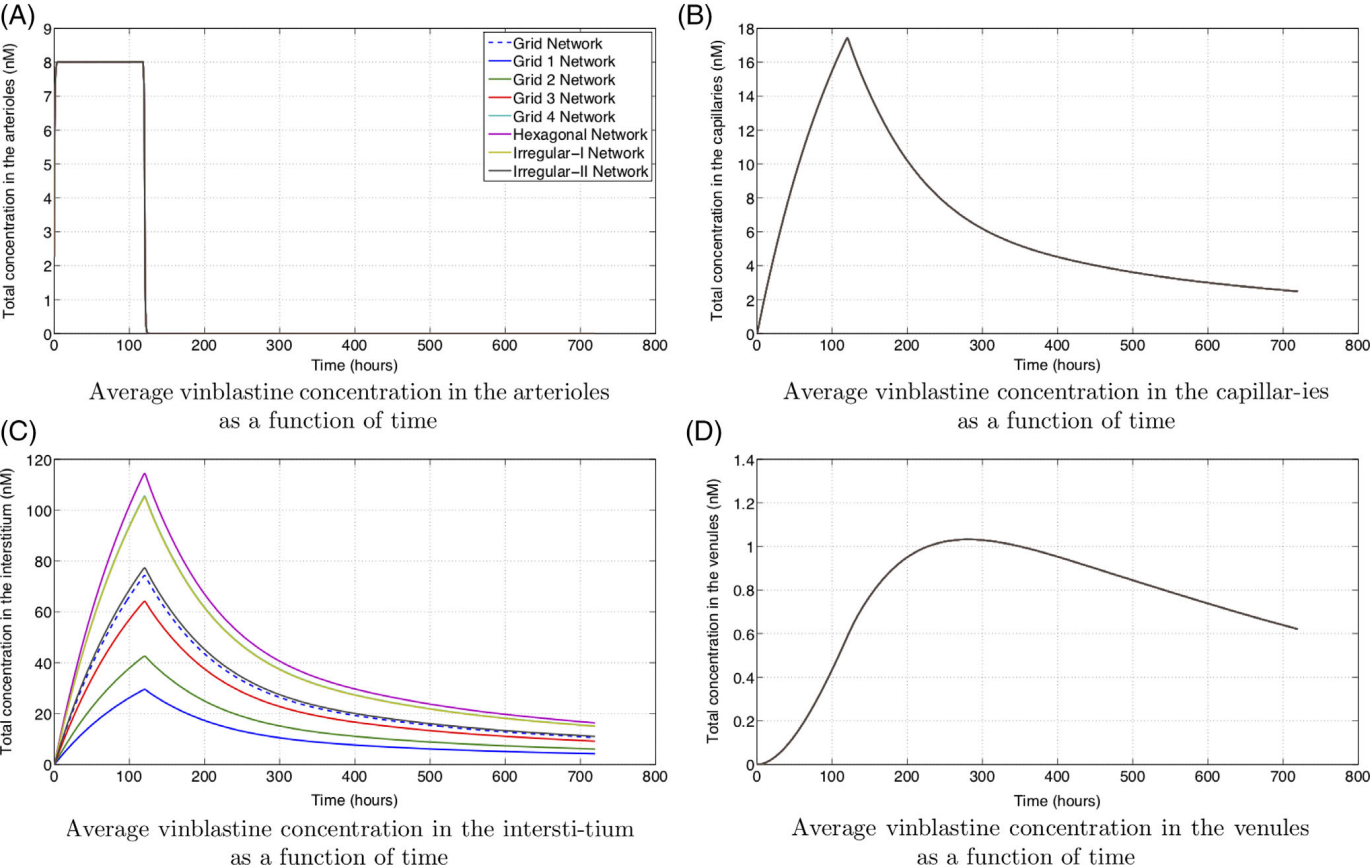
The average concentration in each of the arterioles, capillaries, interstitium, and venules for constant perfusion

#### 2.1.4 Parameter estimation

##### Geometrical parameters

A representative mean interarteriole/venule distance is 10^3^ μm, and the typical size of a dorsal skinfold chamber is 1 cm. This gives a typical value of *η* = *s*/*L* of 10^−1^, justifying the assumption *η* ≪ 1.

##### Physiological parameters

There are two key physiological parameters that are not measured experimentally, to the best of our knowledge; these are *L*_*a*_ and *L*_*v*_, which represent the “leakage” of fluid from the arterioles into the porous tissue matrix and from the porous tissue matrix into the venules, respectively. We define dimensionless parameters *R*_*a*_ and *R*_*v*_ that capture these leakage factors and are defined by *R*_*a*_ = *μL*_*a*_ *L* ^4^/*s* ^5^, *R*_*v*_ = *μL*_*v*_ *L* ^4^/*s* ^5^. Finally, to complete the homogenisation process, we must relate the magnitude of the blood velocity in the capillaries to that in the arterioles or venules. If *V* is a typical blood velocity in the arterioles or venules, then *V* is certainly larger than *U*. We let the ratio *V*/*U* be of size 1/*η* (which is large, as *η* ≪ 1), so that *V*/*U* = *V* ^★^/*η*, where *V* ^★^ = *s* ^4^/*d* ^2^ *L* ^2^ ≈ 4.

#### 2.1.5 Fluid transport on the chamber scale

Finally, we homogenise from the arteriole/venule scale to the chamber length scale to derive the effective model on the length scale of the chamber. The steps here are analogous to those detailed in Shipley and Chapman^18^, ^37^ and also comprise the counterpart to those steps described in Section 2.1.3 for averaging from the microscale to the arteriole/venule scale. This process yields the following model for pressure in the arterioles, venules, capillaries, and interstitium,

(10) $$\nabla \cdot \left(K_{a}\cdot \nabla p_{a}\right)=\frac{\eta R_{a}S_{a}s}{V_{a}}\left(p_{a}-p_{c}\right),\;\nabla \cdot \left(K_{v}\cdot \nabla p_{v}\right)=\frac{\eta R_{v}S_{v}s}{V_{v}}\left(p_{v}-p_{c}\right),$$

(11) $$\nabla \cdot \left(F\cdot \nabla p_{c}\right)=\frac{R_{a}S_{a}s}{V_{p}}\left(p_{c}-p_{a}\right)+\frac{R_{v}S_{v}s}{V_{p}}\left(p_{c}-p_{v}\right),\;p_{t}=p_{c},$$

where the fluid velocities are given by

(12) $$u_{a}=-\frac{1}{\mu }K_{a}\cdot \nabla p_{a},\;u_{v}=-\frac{1}{\mu }K_{v}\cdot \nabla p_{v},\;u_{c}=-\frac{\eta }{\mu }F\cdot \nabla p_{c},\;u_{t}=-\frac{{\nu \eta }^{2}}{\mu }E\cdot \nabla p_{t}.$$

On this length scale of the chamber, the arterioles, venules, capillary bed, and interstitium behave as porous media, with coupled flow between the four. As described above, *S*_*a*_/*V*_*a*_ is the ratio of arteriole surface area to its volume, *S*_*v*_/*V*_*v*_ is the ratio of the venule surface area to its volume, *S*_*a*_/*V*_*p*_ is the ratio of arteriole surface area to porous tissue volume, and *S*_*v*_/*V*_*p*_ is the ratio of venule surface area to porous tissue volume. The parameters *R*_*a*_ and *R*_*v*_ are dimensionless and represent the “leakage” of fluid from the arterioles into the porous tissue matrix and from the porous tissue matrix into the venules, respectively. All parameters are defined in Table 1. The fluid permeability tensors **K**_*a*_, **K**_*v*_, and **F** for the arterioles, venules, and capillaries, respectively, on the length scale of the chamber are given in terms of three cell problems as defined next.

##### Permeability tensors for the interstitial and capillary domains

The fluid permeability tensors **E** and **K** associated with the interstitial and capillary domains are given by

(13) $$E_{\mathrm{ij}}=\delta_{\mathrm{ij}}+\frac{1}{V_{t}}\int_{\text{capillary boundary}}P_{t}^{j}n_{i}\;dS,K_{\mathrm{ij}}=\frac{1}{V_{c}}\int_{V_{c}}w_{\mathrm{ci}}^{j}\;dV,$$

where $P_{t}^{j}$ and $w_{c,i}^{j}$ solve the cell problems

(14) $$\nabla^{2}P_{t}^{j}=0\;\text{in the interstitium},n\cdot \nabla P_{t}^{j}=n\cdot e_{j}\;\mathrm{on}\;\text{the capillary walls},$$

and

(15) $$\nabla \cdot w_{c}^{j}=0,\;\nabla P_{c}^{j}=\nabla_{X}^{2}w_{c}^{j}+e_{j}\;\text{in the capillaries},n\cdot w_{c}^{j}=0,\;w_{c}^{j}\cdot \tau =0\;\mathrm{on}\;\text{the capillary walls}.$$

##### Permeability tensors associated with the arteriolar and venular domains

The fluid permeability tensors **F**, **K** ^*a*^, **K** ^*v*^ are given by

(16) $$F_{\mathrm{kj}}=\left[\delta_{\mathrm{ik}}+\frac{1}{V_{p}}\int_{\text{porous matrix boundary}}P_{p}^{k}n_{i}\;dS\right]K_{\mathrm{ij}},K_{\mathrm{ij}}^{a}=\frac{1}{V_{a}}\int \int \int_{V_{a}}w_{a,i}^{j}\;dV,K_{\mathrm{ij}}^{v}=\frac{1}{V_{v}}\int \int \int_{V_{v}}w_{v,i}^{j}\;dV.$$

The vector **P**_*p*_ solves the cell problem

(17) $$\nabla \cdot \left(K\cdot \nabla P_{p}^{k}\right)=0,$$

in the porous matrix of the arteriole/venule scale, with

(18) $$\left(K\cdot \nabla P_{p}^{k}\right)\cdot n_{i}=-K^{T}\cdot n_{i},$$

for *i* = *a*, *v* on the arteriole/porous matrix and venule/porous matrix boundaries. Additionally, **P**_*p*_ must be periodic and satisfy the uniqueness condition

(19) $$\int \int \int_{V_{p}}P_{p}\;dV=0.$$

Finally, $w_{a}^{j}$ and $w_{b}^{j}$ are solutions of the cell problem,

(20) $$\nabla \cdot w^{j}=0,\;\nabla P^{j}=\nabla^{2}w^{j}+e_{j},\;\text{in the arterioles}/\text{venules with}\;n_{i}\cdot w^{j}=0,\;w^{j}\cdot \tau_{i}=0\;\mathrm{on}\;\text{the boundaries},$$

with **w** ^*j*^ and *P* ^*j*^ periodic on the arteriole/venule length scales.

#### 2.1.6 Discussion

The chamber‐scale model (10) to (12) assumes that fluid flow on the length scale of the chamber is dominated by the arterioles and venules, followed by the capillaries and interstitium in turn (the fluid velocities in the arterioles and venules are largest, whereas those in the capillaries and interstitium are order *η* and order *νη* ^2^ smaller, respectively). The arteriole and venule pressures are coupled to that in the capillaries; indeed, the arteriole and venule pressure appear as sink terms in the capillary pressure Equation (11), whereas the capillary pressure appears as a sink in both the arteriole and venule pressure Equations (10). Finally, the capillary and interstitial fluid pressures are indistinguishable on the length scale of the chamber, but their associated velocities differ because of the different permeability tensors for transport through the capillary bed and interstitium.

The permeability tensors **K**_*a*_, **K**_*v*_, **F**, and **E** can be determined by either comparing predictions of the models (10) to (12) with medical imaging data or by solving explicitly the four unit cell problems (13) to (20) that must be solved on the length scale of the arterioles, venules, and capillaries, once the geometry of these networks has been specified. Essentially, the calculation of **K**_*a*_, **K**_*v*_, **F**, and **E** involves averaging the counterpart local flow variations over their representative unit cell.

We employ this model to investigate the key architectural and functional features that influence fluid perfusion in the capillaries, in a case study relating to a simplified representation of a dorsal skinfold chamber. To simplify the model to one that is sufficiently computationally tractable to explore the impact of key vascular parameters on the model predictions, first of all, we assume that the arteriole and venule networks have identical topological and physiological properties (ie, **K**_*a*_ = **K**_*v*_, *S*_*a*_ = *S*_*v*_, *V*_*a*_ = *V*_*v*_, and *R*_*a*_ = *R*_*v*_), and that all networks are isotropic so that permeability tensors are proportional to the identity matrix. Specifically, we let **K**_*a*_ = **K**_*v*_ = *h* **I**, **F** = (*k*/*V*^★^)**I**, and **E** = (*ks*^2^/*V*^★^)**I**. Here, *V* ^★^ = *η* ^2^/*ν* ^2^ relates the magnitude of the blood velocity in the capillaries to that in the arterioles or venules (specifically if *V* is a typical blood velocity in the arterioles or venules, and *U* is a typical capillary blood velocity, then *V*/*U* = *V* ^★^/*η*), and *κ* = *k* ^*int*^ *s*/*d* ^3^ is the dimensionless interstitial permeability on the length scale of the capillaries. These relationships are a direct consequence of the homogenisation approach. Now, the pressure and velocity equations can be written in terms of parameters that depend purely on the arteriole and venule network and those that depend on the capillary network properties,

(21) $$\nabla^{2}p_{a}=\frac{\mathrm{\eta r}}{\mathrm{hn}}\left(p_{a}-p_{c}\right),\;\nabla^{2}p_{v}=\frac{\mathrm{\eta r}}{\mathrm{hn}}\left(p_{v}-p_{c}\right),\;\nabla^{2}p_{c}=\frac{r}{P}\left(2p_{c}-p_{a}-p_{v}\right),\;p_{t}=p_{c},$$

and

(22) $$u_{a}=-\frac{h}{\mu }\nabla p_{a},\;u_{v}=-\frac{h}{\mu }\nabla p_{v},\;u_{c}=-\frac{\mathrm{\eta P}}{\mu }\nabla p_{c},\;u_{t}=-\frac{\eta^{2}C}{\mu }\nabla p_{t}.$$

Here, *n* and *r* depend purely on the arteriole and venule network properties; *n* is the volume ratio of arterioles (or venules), as a proportion of the volume of the porous tissue, *n* = *V*_*a*_/*V*_*p*_ = *V*_*v*_/*V*_*p*_, whilst *r* depends on the arteriole and venule length scale, *s*, and the chamber length scale, *L*, together with the geometrical and physiological properties of the arteriole and venule networks, through *r* = *R*_*a*_ *sS*_*a*_/*V*_*p*_ = *R*_*v*_ *sS*_*v*_/*V*_*p*_.

The parameter *C* = *k* ^*int*^ *L* ^2^/*s* ^2^ depends on the arteriole and venule length scale, *s*, and chamber length scale, *L*, together the with permeability of the interstitium, *k* ^*int*^. Finally, *P* = *kν* ^2^/*η* ^2^ is the key parameter that depends on the capillary permeability, *k*, and capillary length scale, *d* (note that varying *d* is equivalent to changing the density of the capillary network). The parameter *P* is a measure of the capillary permeability, multiplied by the ratio of the capillary to arteriole with respect to the arteriole to tumour length scales.

### 2.2 Drug transport model

We denote the drug concentration by *c*, with subscripts *a*, *v*, *c*, and *t* denoting the arterioles, venules, capillaries, and interstitium, respectively. Here, we focus on vinblastine, a widely used cell‐cycle specific chemotherapy drug for which the transport kinetics were characterised in Modok et al^38^; however, our models are applicable to any tracer molecule and can be extended to include different drug kinetics. Vinblastine is advected and diffuses in the arterioles, venules, capillaries, and interstitium, and the Péclet number (which represents the ratio of diffusive to convective timescales) is a good way to compare the relative importance of these transport mechanisms. The Péclet number for transport in the capillaries is *Ud*/*D*_*c*_ ≈ 3.8, where *U* ≈ 25 *μ*m s^−1^ is the blood velocity and *D*_*c*_ ≈ 3.3 × 10^−6^ cm^2^ s^−1^ is the diffusion coefficient^38^; therefore, advection and diffusion are in balance on the length scale of the capillaries. On the length scale of the chamber, the Péclet number for transport in the capillaries is *UL*/*D*_*c*_ ≈ 7.6 × 10^3^ indicating that advection dominates over diffusion. Advection also dominates in the arterioles and venules on the length scale of the chamber.

We describe transport from the vessels into the tissue using a membrane law that relates the jump in concentration flux across the membrane, ${\left[J\cdot n\right]}_{-}^{+}$ (where **J** denotes the concentration flux, **n** is the unit outward pointing normal to the vessel/tissue boundary, and “−“ and “+” denote either side of this boundary), to the concentration jump across it, ${\left[c\right]}_{-}^{+}$, via ${\left[J\cdot n\right]}_{-}^{+}=r{\left[c\right]}_{-}^{+}$, where *r* is the boundary permeability (units cm s^−1^).^14^ To give an example, we describe drug transport on the micro‐scale by

(23) $$\frac{\partial c}{\partial t}+\left(u\cdot \nabla \right)c=D\nabla^{2}c,$$

where *D* takes the value *D*_*c*_ within the capillary domain and *D*_*t*_ within the interstitial domain, with

(24) $$\left(c_{c}u_{c}-D_{c}\nabla c_{c}\right)\cdot n-\left(c_{t}u_{t}-D_{t}\nabla c_{t}\right)\cdot n=r\left(c_{c}-c_{t}\right),\;\mathrm{on}\;\text{the capillary walls},$$

subject to an initial condition on the drug concentration field. As for the fluid transport problem reported in Section 2.1, we proceed by first homogenising from the microscale to the arteriole/venule scale and then to the chamber scale to derive an effective model for drug transport. Full details of this process are not reported for succinctness and closely follow the approaches in Shipley and Chapman.^18^, ^37^ The key steps involve (for the example of homogenising from the microscale to the arteriole/venule scale) (a) assuming length scale separation so that **∇** = **∇**_micro_ + *ν* **∇**_arteriole/venule_ in Equations (23) and (24), (b) moving onto the timescale for advection on the arteriole/venule length scale by rescaling *t* = *t*/ν, (c) expanding all variables asymptotically in powers of ν, (c) equating coefficients of powers of ν in (23) and (24), averaging over the microscale to derive the arteriole/venule scale relationships.

This process determines the following transport model for vinblastine on the chamber scale are

(25) $$\frac{\partial c_{a}}{\partial t}+\nabla \cdot \left(c_{a}u_{a}\right)=-\frac{S_{a}T_{a}}{V_{a}}\left(c_{a}-c_{c}\right),\;\frac{\partial c_{v}}{\partial t}+\nabla \cdot \left(c_{v}u_{v}\right)=-\frac{S_{v}T_{v}}{V_{v}}\left(c_{v}-c_{c}\right),$$

(26) $$\frac{\partial c_{c}}{\partial t}+\nabla \cdot \left(c_{c}u_{c}\right)=\frac{S_{a}T_{a}}{V_{p}}\left(c_{a}-c_{c}\right)+\frac{S_{v}T_{v}}{V_{p}}\left(c_{v}-c_{c}\right),$$

where the fluid velocities **u**_*a*_, **u**_*v*_, and **u**_*c*_ are given by (22), and *T*_*i*_ for *i* = *a*, *v* are coefficients given by *T*_*a*_ = *r*_*a*_/*η* ^2^, *T*_*v*_ = *r*_*v*_/*η* ^2^ that represent drug transport from the arterioles (or venules) into the porous tissue matrix composed of capillaries and interstitium. The drug concentration in the interstitium and capillaries is indistinguishable on the chamber scale so that *c*_*t*_ = *c*_*c*_. This is a direct consequence of the fact that (a) advection and diffusion of vinblastine are balanced in the capillaries and interstitium (the concentration of vinblastine is well‐mixed over the distances relevant here) and (b) vinblastine is a tracer, so there is no uptake to introduce spatial gradients in concentrations.

## 3 FLUID TRANSPORT RESULTS

We investigate the impact of varying the properties of the capillary bed on fluid perfusion in a case study of a simplified geometry representative of a dorsal skinfold chamber, by solving the fluid transport Equations (21) and (22) using the finite element package COMSOL Multiphysics. To achieve this, we fix the geometry of the chamber, the topology and physiological properties of the arteriole and venule networks (ie, the parameters *n* and *r*), and the interstitial permeability (ie, the parameter *C*) and test the impact of varying the capillary configuration, which explicitly means varying the parameter *P*.

Experimentally chambers are imaged in 2D slices through their centre, so we simplify the investigation by assuming a 2D set‐up. The chamber is represented in 2D by a square of dimensions *L* cm × *L* cm with a central circle of cancerous tissue (region 1), as depicted in Figure 6. A source artery and sink vein are seeded in region 1 and are represented by a circle, where the upper half‐circle is the artery, and the lower half‐circle is the vein (this mimics the typical experimental scenario where the tumour mass is connected to the host blood supply through seed ing an artery and vein into the region). The remainder of the chamber is composed of malignant tissue (region 2). Throughout the chamber, we solve Equation (21) for the arteriole and venule pressures; we impose a pressure drop across the vasculature by applying *p*_*a*_ = 50 mmHg on the artery boundary and *p*_*v*_ = 10 mmHg on the vein boundary. We also apply no flux of *p*_*a*_ and *p*_*v*_ across the outer boundary of the chamber and no flux jump across the interface between regions 1 and 2. In region 2, we fix the value of *P* at *P*_2_ = *k*_2_ *L* ^2^ *d*_2_^2^/*s* ^4^. We impose no flux of *p*_*c*_ through the outer boundary of the chamber and no flux jump across any internal boundaries. Finally, for simplicity, we assume that the arteriole, venule, and capillary networks in region 2 each have a hexagonal structure, as shown in Figure 8B. This allows us to test the perfusion sensitivity in response to variation in the capillary structure in region 1. Table 1 provides a summary of the fixed parameters.

To test the impact of varying *P* on fluid perfusion in the capillaries and interstitium, we evaluate the flux of fluid in the capillaries and interstitium that travels from region 1 into region 2,

(27) $$Q_{c}=\int u_{c}\cdot ndS=-\frac{\eta }{\mu }P\int_{\Gamma }\nabla p_{c}\cdot ndS,\;Q_{t}=\int u_{t}\cdot ndS=-\frac{\eta^{2}C}{\mu }\int_{\Gamma }\nabla p_{t}\cdot ndS=\frac{\mathrm{\eta C}}{P}Q_{c},$$

where Γ is the interface between both regions and **n** is the unit outward pointing normal to Γ.

The results presented here are for a fixed value of *R*_*a*_ = *R*_*v*_ = 10^−2^. These parameters characterise the leakage of fluid from the arterioles and venules into the capillary bed and are not measured experimentally. Model outcomes are only sensitive to the value of *R*_*a*_ = *R*_*v*_ when this leakage coefficient is small (representing the physiologically unrealistic situation of the arterioles and venules short‐circuiting the capillary bed). Otherwise, model predictions are much more sensitive to the value of *P*, which captures the capillary bed properties.

Figure 5 shows how the fluxes *Q*_*c*_ and *Q*_*t*_ vary as a consequence of changes in the permeability and density of the region 1 capillary network (through *P*). For the smallest value of *P* tested (*P* = 10^−16^ m^2^), *Q*_*c*_ and *Q*_*t*_ are small and positive, corresponding to a net flux of fluid from region 1 to region 2. As *P* increases, so do *Q*_*c*_ and *Q*_*t*_ until maxima are reached when *P* ≈ 1.25 × 10^−12^ m^2^ (here, *Q*_*c*_ = 1.45 × 10^4^ *μ*m^2^ s^−1^ and *Q*_*t*_ = 6.18 × 10^3^ *μ*m^2^ s^−1^, respectively). As *P* now increases from 1.25 × 10^−12^ m^2^, *Q*_*c*_ and *Q*_*t*_ decrease and are negative for *P* > 5.2 × 10^−6^ m^2^, corresponding to a net flux of fluid from region 2 to region 1.

Figure 6 shows the arteriole and venule fluid pressures throughout the chamber for an example value *P* = 10^−14^ m^2^. Given that the arteriole and venule properties are fixed, arteriole and venule pressure distributions are similar for all values of *P*; however, the precise pressure drop across the arteriole and venous networks does vary as the underlying capillary bed properties are varied (results not shown).

Figure 7A shows the capillary pressure distribution when *P* = 10^−14^ m^2^. This low value of *P* corresponds to either a low capillary network permeability, *k*, or high density (through the capillary length scale *d*) in region 2. The capillary pressure is highest (or lowest) in the immediate vicinity of the source artery (or sink vein) but decays relatively quickly to a background value of around 31 mmHg. Indeed, the capillary pressure in most of the chamber is approximately constant. This results in gradients in *p*_*c*_ from the immediate vicinity of the source artery/sink vein and the consequent positive values of *Q*_*c*_ and *Q*_*t*_ at this value of *P*.

Figure 7B shows the pressure distribution for *P* = 1.25 × 10^−12^ m^2^, corresponding to the maxima of *Q*_*c*_ and *Q*_*t*_ (see Figure 5). The spatial gradients in the capillary pressure throughout the chamber are larger, resulting in larger capillary and interstitial fluid velocities and the consequent high values of *Q*_*c*_ and *Q*_*t*_. This increase in *P* relative to Figure 7A could be achieved by either increasing the permeability of the region 1 capillary network or decreasing its density.

As *P* increases from 1.25 × 10^−12^ m^2^, *Q*_*c*_ and *Q*_*t*_ progressively decrease and are negative for *P* > 5.2 × 10^−12^ m^2^, corresponding to a net flux of fluid from region 2 to region 1. The consequence of increasing *P* (from 1.25 × 10^−12^ m^2^) on the pressure distributions can be seen in Figures 7C‐E. As a result of the asymmetrical position of the seeded artery and vein (and the value of *P* relative to *P*_2_ = 9.36 × 10^−12^ m^2^), as *P* increases, a localised region of high/low capillary pressure develops in the top/bottom left hand corner of the chamber and the capillary pressure in region 1 approaches constant. Therefore, although there is a net flow from the region 2 to region 1, the capillary and interstitial fluid velocities within region 1 become very small, and drugs will accumulate in the tumour periphery as opposed to being transported throughout the tumour.

Larger capillary and interstitial velocities in region 2 are beneficial to distribute drugs by advection to the cancerous cells. This analysis indicates that an optimal *P* value of 1.25 × 10^−12^ m^2^ will achieve this and can be realised by varying the permeability or density of the capillary bed. For example, if the *P* value of region 1 is larger than 1.25 × 10^−12^ m^2^, vascular intervention that reduces the *P* value would benefit drug distribution and could be achieved by either reducing the permeability or increasing the density of the capillary network. Similarly, if *P* < 1.25 × 10^−12^ m^2^, vascular intervention that increases the *P* value would benefit drug distribution.

Finally, we investigate some explicit network examples. We test eight different structures for the region 1 capillary network; the unit cells for these networks are shown in Figure 8. Figure 8A,B are two examples of regular networks (a grid and hexagonal structure, motivated by the honeycomb structures imaged in Konerding et al^28^), whereas the remaining six structures are more irregular (the networks in Figure 8E‐H have identical inlets and outlets to the grid network and are formed by progressively removing one link of the network each time). Figure 9 shows the values of the permeability tensor **F** = *F*_*ij*_ (μm^2^), which are determined by solving the full cell problems given in Section 2.1.5 using COMSOL Multiphysics. We assume that the arteriole and venule networks, and the region 2 capillary network, take the hexagonal structure of Figure 8B. We also take *d* = 50 *μ*m in both regions.

The *P* values and corresponding values of *Q*_*c*_ and *Q*_*t*_ for each network are summarised by bar charts in Figures 9. The *P* values for these networks lie in the range 9.52 × 10^−12^ m^2^ to 6.98 × 10^−10^ m^2^ and increase from smallest to largest in the order Hexagonal, Irregular‐II, Irregular‐I, Grid, Grid‐III, Grid‐I, Grid‐II, Grid‐IV (see Table 2). If we exclude the Hexagonal case, all *P* values lie in the range 3.13 × 10^−11^ m^2^ to 6.98 × 10^−10^ m^2^ and correspond to virtually constant capillary pressure in region 1. These values are larger than the optimal value of *P* = 1.25 × 10^−12^ m^2^ identified earlier, indicating that vascular intervention to decrease the *P* value would benefit anticancer drug distribution throughout region 1. This decrease in *P* value could be achieved by either decreasing the permeability of the networks (ie, decreasing the matrix components *F*_*ij*_) or by increasing the density of the capillary bed by decreasing *d*.

**Table 2:** The values of *V*_*t*_/*V*_*c*_ for each explicit network under consideration

Network	Grid‐I	Grid‐II	Grid‐III	Grid	Irregular‐II	Irregular‐I	Grid‐IV	Hexagons
*V*_*t*_/*V*_*c*_	1.6933	2.4458	3.6812	4.2632	4.4358	6.0295	6.0571	6.5651

## 4 VINBLASTINE DISTRIBUTION RESULTS

Vinblastine is a chemotherapeutic drug frequently used to treat various cancers via intravenous delivery. As its properties are well‐characterised,^39^ we next investigate vinblastine perfusion in a dorsal skinfold chamber, by solving the equations for vinblastine transport given by Equations (25) and (26) using the finite element package COMSOL Multiphysics. To facilitate the computation, Equations (25 and (26) are solved with an additional numerical diffusion constant of value $O\left(\eta^{2}\right)$ in keeping with the next order corrections to the transport model, which are neglected. Vinblastine is delivered exclusively intravenously to a patient using either (a) a single injection or (b) constant perfusion of the drug to the patient over a long time period. These correspond to two different boundary conditions for the arteriole concentration, *c*_*a*_, on the artery wall. The functional form for this boundary condition is denoted by *σ* and discussed in more detail below. On the external boundaries of the chamber, we apply no flux condition for each concentration component, with no flux jump on the internal boundaries.

### 4.1 Treatment through a single injection

A dose of drug delivered by injection will be metabolised in the bloodstream as time progresses. For vinblastine, this metabolism occurs over four phases,^39^ namely, an initial fast phase that represents the distribution of the blood through the body and three further slow phases representing the redistribution and metabolism of the drug in different organs of the body. This can be represented mathematically by the function

(28) $$\sigma \left(t\right)=Ae^{-k_{1}t}+Be^{-k_{2}t}+Ce^{-k_{3}t}+De^{-k_{4}t},$$

where the *k*_*i*_ (*i* = 1, 2, 3, 4) represent the half lives of the four separate phases, and *A*, *B*, *C*, and *D* are constants to be determined for a specific individual. The half lives for the four distribution/metabolism phases are 1, 4, 53, and 1173 seconds, respectively, giving *k*_1_ = 41.6 min^−1^, *k*_2_ = 0.17 min^−1^, *k*_3_ = 1.3 × 10^−2^ min^−1^, and *k*_4_ = 5.9 × 10^−4^ min^−1^.

The constants *A*, *B*, *C*, and *D* are determined from the initial dose, together with three conditions on the level of metabolism after each phase's half life.^39^ We consider an initial dose of 2700nM; for a person of weight 64 kg (and blood volume ≈ 4.4 L), the remaining conditions are *σ*(*t* = 4 min) = 700 nM, *σ*(*t* = 53 min) = 150 nM, and *σ*(*t* = 1173 min) = 10 nM, which give *A* = 1557nM, *B* = 862nM, *C* = 261nM, and *D* = 20nM.

### 4.2 Treatment through constant perfusion

An alternative regime involves maintaining a constant concentration of vinblastine in the bloodstream over a longer time period. To limit damage to the patient, the achievable concentration is lower than the initial injection concentration; indicative values are a concentration of 8nM applied constantly to the patient over a period of 5 days.^40^ The boundary condition on the artery wall is therefore

(29) $$\sigma \left(t\right)=\left\{\begin{array}{cc} 8\mathrm{nM} & \text{for}\;0\leq t\leq 120\;\text{hours}\\ 0 & \text{for}\;t>120\;\text{hours} \end{array}\right..$$

Next, we solve Equations (25) and (26) to test whether treatment through a single injection or constant perfusion is more efficient at killing tumour cells in a dorsal skinfold chamber. To the best of our knowledge, there are no data available on *r*_*a*_ and *r*_*v*_, which represent the timescale for vinblastine transfer between the arterioles and capillary bed and between the capillary bed and venules. Here, we fix *r*_*a*_ = *r*_*v*_ = 23.1 × 10^−12^ s^−1^ based on our order of magnitude estimates using homogenisation theory. Varying *r*_*a*_ = *r*_*v*_ influences the time delay before the maximum value of the capillary/interstitial concentration, together with the values at the peak. It does not, however, alter the qualitative conclusions that will be made.

We evaluate the cell kill rate as a consequence of the vinblastine treatment. The maximum kill rate is 1/24 h^−1,^ 41 whereas the concentration of vinblastine required to kill cells at the half‐maximal kill rate is 2nM.^42^ Finally, the cell kill rate is proportional to concentration for low drug concentrations so we assume that the cell‐kill rate, $M_{c}\left(c_{t}\right)=\frac{\rho_{1}c_{t}}{\rho_{2}+c_{t}}$, where *ρ*_1_ = 1/24 h^−1^, *ρ*_2_ = 2nM.

We test the impact of the cell‐kill rate on the interstitial volume fraction by modelling the time‐dependent changes in this volume fraction using a partial differential equation, where we explicitly focus on the competition between cell proliferation and death, with the latter determined as a function of the local concentration of drug. This serves as a first approximation to the impact of the treatment therapy, as there is no mechanism for volume change because of tumour growth or regression in the models presented here, but provides an illustrative example of the utility of the model. We denote the interstitial volume fraction by *ϕ* and capture changes in this volume fraction through

(30) $$\frac{\partial \phi }{\partial t}\left(x,t\right)=\mathrm{K\phi}\left(x,t\right)-M_{c}\left(c_{t}\left(x,t\right)\right)\phi \left(x,t\right),$$

subject to the initial condition *ϕ*(**x**, 0) = *n*_*t*_, where *K* is the net cell proliferation rate and depends on the cell line used. We note that Equation (30) assumes that the cell proliferation and cell kill rates are proportional to the interstitial volume fraction, following similar approaches in the literature. Finally, we assess cell kill by evaluating the spatial average of *ϕ*, given by

(31) $$\phi^{\mathrm{av}}\left(t\right)=\frac{1}{\text{Area of Region}\;1}\int_{\text{Region}\;1}\phi \left(x,t\right)dV,$$

where the area of the region 1 is 0.4948 cm^2^ for the simulations in this paper. We present results for three cases of the cell proliferation rate *K* = 1/29, 1/31, 1/33 h^−1^.

The individual average vinblastine concentrations in the arterioles, venules, capillaries, and interstitium are shown in Figures 11 and 12. For both treatments through injection or constant perfusion, the concentrations in the arterioles closely mimic the treatment profile. Variation due to the capillary structure in region 1 is barely detectable as the timescale for vinblastine transport is much faster than that for uptake or proliferation. Indeed for constant perfusion, the vinblastine concentration is constant (to three significant figures) across the entire chamber within 3.5 hours (the simulation length is 712 hours).

The value of *ϕ* ^*av*^ increases and decreases from the initial value because of the balance of cell kill and proliferation, as described by Equations (30) and (31). Given that the concentration of vinblastine is uniform throughout the chamber after about 3.5 hours, the only dependence of *ϕ* ^*av*^ on vascular structure occurs through the initial condition *ϕ*(**x**, 0) = *n*_*t*_. A comparison of treatment through single injection and constant perfusion is shown in Figure 10 for three different cases of the cell proliferation rate *K* = 1/29, 1/31, 1/33 h^−1^. The concentration of vinblastine delivered to the chamber is much lower for constant perfusion than for a single injection but is maintained for a longer period of time. This does not appear to have a significant cumulative effect. For example when the rate of cell proliferation *K* = 1/29 hours^−1^, the final values of *ϕ* ^*av*^ were in the range (1.4936, 2.0616) for a single injection and the range (9.2587, 12.7797) for constant perfusion. This pattern of behaviour is replicated for other values of the rate of cell proliferation, *K*.

The modelling outputs can also predict the timing of a second injection of vinblastine. This decision could be made based on various criteria such as when the value of *ϕ* ^*av*^ reaches a minimum value, or when the vinblastine concentration in the capillaries drops to a prescribed value. For example, when the cell proliferation rate *K* = 1/31 h^−1^, the minimum value of *ϕ* ^*av*^ is achieved after 14.8 days and could motivate a 2‐week cycle of vinblastine treatment. We note that this framework neglects multiple features necessary to quantitatively predict drug treatment efficacy (eg, tumour growth and toxicity); however, this case study does enable the behaviour of the new four‐compartment model to be explored. We also note that a much more marked dependence on vascular architecture would be expected for drugs that decay and are metabolised, because of the impact of spatial gradients in the drug profile at the scale of the capillaries.

## 5 CONCLUSIONS

We have presented a new four‐component model to investigate fluid and vinblastine perfusion in a dorsal skinfold chamber. The model incorporated arteriole, capillary, and venule networks, together with the interstitium, and provides a framework for incorporating the impact of these hierarchical structures on fluid and drug perfusion to tumours. The impact of treatment through a single injection and through constant perfusion was tested, and the dependence of both the fluid and vinblastine perfusion results on the vascular network was highlighted.

The multiscale model of blood transport was used to identify a single parameter (*P* in this paper), which relates chamber‐scale fluid perfusion to the capillary bed properties (specifically the permeability and density of the capillary network). By fixing the topological and physiological properties of the arteriole and venules networks, a *P* value of 1.25 × 10^−12^ m^2^ was identified that maximises fluid perfusion through the tumour region of the chamber.

The multiscale model of vinblastine transport was used to explore the effectiveness of treatment via a single injection or constant perfusion of the drug, when the cancer cell proliferation rate in chamber was *K* = 1/29, 1/31, 1/33 h^−1^. In each case, treatment through injection was significantly more effective than that through constant perfusion, although we note the necessity for more complexity to be incorporated, and model validation, before these predictions could be taken forward. In addition, it was demonstrated how to use the model outputs to predict when to deliver a second course of chemotherapy.

There are numerous opportunities for further extension of the approach in this paper, spanning incorporating more complexity into the modelling frameworks, and validation of the model predictions against real world data. The current work focuses on the development of a new four‐component model that incorporates relevant transport information on a hierarchy of length scales and its implementation in a case study to explore fluid and drug perfusion in a relevant experimental set‐up. Further opportunities for model development include extension to realistic, 3D vascular geometries and tumour architectures acquired using medical imaging, including more realistic haemodynamic descriptions at the capillary scale, the incorporation of more sophisticated drug kinetics (eg, decay and metabolism, which will involve a different homogenised model), and also tumour growth. There is an outstanding need for more sophisticated descriptions of tumour perfusion, growth, and response to treatment to inform therapy developments for cancer. This would require extensive validation of the assumptions and predictions of the model against measurable data. We leave these as important next steps to develop the utility of this approach in informing tumour treatment strategies.

## CONFLICT OF INTEREST

None.

## GLOSSARY OF TERMS

**Dimensional Parameters**
*d*: Intercapillary separation *μ*m
*s*: Interarteriole and intervenule separations *μ*m
*L*: Chamber length scale cm
**u**: Fluid velocity cm s^−1^
*p*: Fluid pressure mmHg
**K**_*a*_ and **K**_*v*_: Arteriole and venule fluid permeability tensors m^2^
**F** and **E**: Capillary and interstitial fluid permeability tensors m^2^
*S*_*a*_ and *S*_*v*_: Arteriole and venule surfaces areas in contact with porous tissue matrix m^2^
*V*_*a*_, *V*_*v*_ and *V*_*p*_: Arteriole, venule and porous tissue matrix volumes m^3^
*μ*: Viscosity of blood Pa s
*h*: Isotropic component of arteriole and venule permeability tensors m^2^
*k*: Isotropic component of capillary permeability tensor m^2^
*k*^*int*^: Interstitial permeability m^2^
*P* = *kν*^2^/*η*^2^: Parameter that captures the region 2 capillary network properties m^2^
*C* = *k*^*int*^/*η*^2^: Parameter that captures the interstitial permeability m^2^
*c*: Vinblastine concentration nM
*U*: Typical capillary velocity μm s^−1^
*D*_*c*_: Vinblastine diffusivity in the capillaries cm^2^ s^−1^
*r*: Membrane permeability to vinblastine transport s^−1^
*T*_*a*_ = *r*_*a*_/*η*^2^: Parameter representing drug transport from the arterioles into the porous tissue matrix s^−1^
*T*_*v*_ = *r*_*v*_/*η*^2^: Parameter representing drug transport from the arterioles into the porous tissue matrix s^−1^
*Q*_*c*_: Flux of fluid in the capillaries from region 1 to region 2 μm^2^ s^−1^
*Q*_*t*_: Flux of fluid in the interstitium from region 1 to region 2 μm^2^ s^−1^
*σ*(*t*): Vinblastine treatment boundary condition on the artery wall nM
*M*_*c*_: Functional form for cell kill rate due to vinblastine nM hr^−1^
*K*: Net cell proliferation rate s^−1^

**Dimensionless ratios**
*ν* = *d*/*s*: Ratio of capillary to arteriole/venule length scales ‐
*η* = *s*/*L*: Ratio of arteriole/venule to chamber length scales ‐
*R*_*a*_ and *R*_*v*_: Dimensionless parameters that represent fluid “leakage” from the arterioles and venules into the porous tissue matrix ‐
*n* = *V*_*a*_/*V*_*p*_ = *V*_*v*_/*V*_*p*_: Volume ratio of arterioles (and venules) as a proportion of the volume of the porous tissue ‐
*r* = *R*_*a*_*sS*_*a*_/*V*_*p*_ = *R*_*v*_*sS*_*v*_/*V*_*p*_: Dimensionless parameter that depends on the geometrical and physiological properties of the arteriole and venule networks ‐
*n*_*a*_: Volume fraction of arterioles ‐
*n*_*v*_: Volume fraction of venules ‐
*n*_*p*_: Volume fraction of porous tissue matrix ‐
*n*_*c*_: Volume fraction of capillaries ‐
*n*_*t*_: Volume fraction of interstitium ‐
*ϕ*(**x**, *t*): Interstitial volume fraction ‐
*ϕ*(*t*)^*av*^: Volume‐averaged interstitial volume fraction ‐
